# MEVALONATE KINASE represses anthocyanin biosynthesis via sucrose transporters and gibberellin synthesis pathways in Arabidopsis

**DOI:** 10.21203/rs.3.rs-8124382/v1

**Published:** 2025-12-31

**Authors:** Jinku Kang, Sua Cho, Kiyoon Kang, Daewon Kim, Sang-Il Bae, Eunji Shin, So-Yon Park, Gary Stacey, Nam-Chon Paek, Sung-Hwan Cho

**Affiliations:** Seoul National University Agriculture and Life Sciences Library: Seoul National University College of Agriculture and Life Sciences; Seoul National University Agriculture and Life Sciences Library: Seoul National University College of Agriculture and Life Sciences; Incheon National University; Gyeongsang National University; Seoul National University Agriculture and Life Sciences Library: Seoul National University College of Agriculture and Life Sciences; Seoul National University College of Agriculture and Life Sciences; University of Missouri; University of Missouri; Seoul National University College of Agriculture and Life Sciences; Chungbuk National University

**Keywords:** Anthocyanin, Arabidopsis thaliana, Gibberellic acid, Mevalonate pathway, MVK, SUC1

## Abstract

Anthocyanins, a class of flavonoid pigments, function as crucial modulators of plant responses to environmental stressors by mitigating oxidative damage and facilitating cellular adaptation. Anthocyanin biosynthesis is tightly regulated by transcriptional networks that respond to developmental cues and external stimuli. Here, we identify MEVALONATE KINASE (MVK), a key enzyme of the cytosolic isoprenoid biosynthesis pathway, as a repressor of sucrose-induced anthocyanin production in Arabidopsis. Loss-of-function *mvk* mutants show increased anthocyanin levels compared to wild-type (WT) plants under high sucrose conditions. The expression of anthocyanin biosynthetic and regulatory genes, such as *CHS, DFR*, and *MYB75/PAP1*, is increased in *mvk-1* mutants grown in the presence of high sucrose. *mvk-1* mutants exhibited elevated sucrose accumulation through upregulation of sucrose transporters compared to WT under high sucrose conditions. Furthermore, reduced levels of gibberellins in *mvk-1* mutants resulted in the stabilization of DELLA proteins, which are known repressors of gibberellin signaling, thereby facilitating sucrose-induced anthocyanin accumulation. Our findings demonstrate that MVK negatively regulates sucrose-induced anthocyanin biosynthesis by modulating sucrose transport and gibberellin homeostasis in Arabidopsis.

## Introduction

Anthocyanins, a pivotal class of water-soluble flavonoids, are responsible for pigmentation of vegetative (leaves, stems, and roots) and reproductive (flowers and fruits) organs in plants. These pigments are not only aesthetic, but also serve crucial ecological functions, such as attracting pollinators and seed dispersers, which are essential for reproduction and survival (Harborne and Williams 2000). Beyond their ecological roles, anthocyanins are widely recognized for the role in abiotic stress responses, particularly through antioxidant properties that mitigate oxidative damage caused by reactive oxygen species (ROS) (Grotewold 2006; Xu et al. 2017). Additionally, anthocyanins offer nutritional benefits to humans, making anthocyanin-rich plants increasingly important research targets (Khoo et al. 2017).

The biosynthesis of anthocyanins is regulated by a combination of developmental cues and environmental signals, including light, temperature, and several endogenous molecules (LaFountain and Yuan 2021). Among them, sucrose is known to promote anthocyanin biosynthesis (Solfanelli et al. 2006). In addition, phytohormones, including ethylene, jasmonic acid (JA), gibberellic acid (GA), abscisic acid (ABA), and cytokinin (CK), interact with sucrose signaling pathways, collectively modulating anthocyanin production (Das et al. 2012; Loreti et al. 2008).

Anthocyanins are synthesized in the cytosol, then modified into various derivatives and subsequently transported into vacuoles. In Arabidopsis (*Arabidopsis thaliana*), structural genes involved in anthocyanin biosynthesis are classified into early biosynthetic genes (EBGs) and late biosynthetic genes (LBGs) (Deroles 2009). Briefly, the pathway starts with condensation of one molecule of 4-coumaroylcoenzyme A and three molecules of malonyl-CoA leading to the formation of naringenin chalcone. EBGs such as chalcone synthase (CHS), chalcone isomerase (CHI), flavonol 3-hydroxylase (F3H), and flavonol 3-hydroxylase (F3′H) further metabolize naringenin chalcone leading to the production of flavonols. Subsequently, LBGs, including dihydroflavonol-4-reductase (DFR), leucoanthocyanidin dioxygenase (LDOX), anthocyanidin reductase (ANR), and UDP-Glc:flavonoid 3-O-glucosyltransferase (UF3GT) facilitate the final steps that produce anthocyanins and proanthocyanidins (Holton and Cornish 1995).

Anthocyanin biosynthesis is tightly regulated at the transcriptional level by the MBW (MYB-bHLH-WD40) complex, which consists of R2R3-MYB, bHLH, and WD40-repeat proteins (Hichri et al. 2011). In Arabidopsis, the MBW complex includes R2R3-MYBs (MYB75/PAP1, MYB90/PAP2, MYB113, and MYB114), bHLH transcription factors [TRANSPARENT TESTA 8 (TT8), GLABRA 3 (GL3), and ENHANCER OF GLABRA 3 (EGL3)], and the WD40-repeat protein TRANSPARENT TESTA GLABRA 1 (TTG1) (Broun 2005; Gonzalez et al. 2008). This complex orchestrates anthocyanin biosynthesis by activating the expression of structural genes such as *Phenylalanine ammonia-lyase* (*PAL*), *CHS, CHI, F3H, DFR, ANS*, and *UF3GT*, ultimately promoting anthocyanin accumulation in vegetative and reproductive tissues (Das et al. 2012). Recent studies in Arabidopsis showed that GLK1 promotes anthocyanin accumulation by directly interacting with MYB75, MYB90, and MYB113, thereby boosting their transcriptional activity (Li et al. 2023). In addition, energy deficiency suppresses anthocyanin accumulation through the action of SnRK1, a master metabolic regulator. SnRK1 destabilizes MYB75, thus repressing MBW-mediated transcription and anthocyanin production under low energy conditions (Broucke et al. 2023). Homologs such as FvMYB10, FvMYB41, and FvMYB105 interact with bHLH partners like FvbHLH33 and FvMYC1 to regulate the synthesis of stage-specific anthocyanin and proanthocyanidin during fruit ripening in woodland strawberry (Xu et al. 2021). Importantly, the MBW activity is subject to modulation by internal and external signals, such as hormones and sugar. DELLA proteins, mostly acting as repressors of gibberellin (GA) signaling, promote anthocyanin biosynthesis by sequestering JAZ and MYBL2 repressors, thereby enabling MBW activation (Xie et al. 2016). In addition, sucrose enhances anthocyanin biosynthesis by stabilizing DELLA proteins and inducing expression of MBW-regulated genes such as *MYB75/PAP1, CHS*, and *DFR* (Li et al. 2014).

Given its dual role as both a carbon source and a signaling molecule, sucrose not only provides metabolic substrates but also functions as a key regulator that integrates hormonal and transcriptional networks to promote anthocyanin accumulation (Loreti et al. 2008; Sakr et al. 2018). Sucrose content in plants may increase due to alterations in sucrose metabolism or the activity of sucrose transporters (Julius et al. 2017). Sucrose transporters (SUCs) are sucrose-proton symporters involved in sucrose translocation (Braun 2022). Nine putative *SUC* genes have been identified in Arabidopsis, and some are directly linked to anthocyanin accumulation. For example, SUC1 plays a critical role in sucrose-induced anthocyanin accumulation, and sucrose-induced anthocyanin accumulation is inhibited in *SUC1* knockout mutants (Sivitz et al. 2008). In addition, the Arabidopsis *pho3* mutants, which is defective in SUC2 function, exhibits enhanced anthocyanin accumulation (Lloyd and Zakhleniuk 2004). Among *SUC* genes, *SUC5* is expressed specifically in the endosperm, *SUC6* and *SUC7* encode aberrant proteins, and *SUC8* and *SUC9* are predominantly expressed in floral tissues (Baud et al. 2005; Meyer et al. 2000; Sauer et al. 2004).

The mevalonate (MVA) pathway, functioning in the cytosol of plant cells, plays a crucial role in primary metabolism by producing isoprenoids, sterols, and other key metabolites (Pulido et al. 2012). It is conserved across plants, fungi, and animals and supports diverse physiological processes (Miziorko 2011; Ruiz-Sola et al. 2016; Yang et al. 2021). Within this pathway, mevalonate kinase (MVK) is a key enzyme that phosphorylates mevalonic acid to produce mevalonate 5-phosphate (Riou et al., 1994). A recent study showed that Arabidopsis MVK is a direct phosphorylation target of P2K1, leading to activation of the MVA pathway in response to extracellular ATP (eATP) elicitation (Cho et al. 2022). The relationship between the MVA pathway and anthocyanin production has been studied in apple trees (*Malus domestica* Borkh). This pathway produces isoprenoids and sterols and was shown to influence anthocyanin accumulation by positively regulating IAA and ABA synthesis while inhibiting GA synthesis (Flores-Perez et al. 2010; Li et al. 2018). However, the mechanism of MVA-mediated anthocyanin regulation in plants remains to be elucidated.

In this study, we aimed to elucidate the role of MVK, a core enzyme of the cytosolic isoprenoid biosynthesis pathway, in the regulation of sucrose-induced anthocyanin biosynthesis in Arabidopsis. Although the MVA pathway was previously associated with various metabolic and hormonal signaling events, its connection to anthocyanin production in response to sucrose remains poorly understood. Our findings reveal that *mvk-1* mutants accumulate more anthocyanins than WT under high sucrose conditions. This phenotype is accompanied by elevated gene expression of anthocyanin biosynthesis such as *CHS* and *DFR*, and transcriptional regulators such as *MYB75/PAP1*. Furthermore, we demonstrate that MVK negatively regulates anthocyanin accumulation through two distinct mechanisms. First, MVK inhibits the expression of *SUC1*. Genetic analysis of *mvk-1 suc1–5* double mutants further revealed that this regulation is mediated by a SUC1-dependent regulatory pathway. Second, mutation of *MVK* reduces gibberellin levels, thereby promoting stabilization of DELLA protein. Collectively, our results uncover a previously uncharacterized function of MVK as a negative regulator of sucrose-induced anthocyanin biosynthesis, integrating sugar transport and hormonal signaling into a coordinated regulatory network.

## Materials and methods

### Plant material and growth conditions

Wild-type aequorin-expressing transgenic Arabidopsis ColQ (Col-0 background) plants were provided by Marc Knight (Knight et al. 1996). The *mvk-1* mutant (ColQ background) has been described previously (Cho et al. 2022). Arabidopsis seeds were surface-sterilized and sown on half-strength Murashige and Skoog (MS) medium supplemented with 0% (w/v) sucrose, 0.5% (w/v) agar (MB Gellan Gum, Cat No. MB-G4367), and 0.05% (w/v) MES (pH 5.7). Following a 3-day cold stratification at 4°C, the plates were positioned vertically in a growth chamber set to a 16 h light/8 h dark photoperiod at 22°C, and 100 μmole m^−2^ s^−1^ light intensity.

### Generating CRISPR-Cas9

Generating CRISPR-Cas9

The *suc1–5* mutant was generated via CRISPR/Cas9-mediated genome editing, with a single guide RNA (sgRNA, 5−CTCGATℂCTGGGACA⊤ℂTGG−3) targeting the SUC1 coding region being designed using the CRISPR direct program (http://crispr.dbcls.jp/) (Naito et al. 2015). The tRNA–gRNA–Cas9 fragment was inserted into the *pRGEB32* vector (Xie et al. 2015). This binary vector was transformed into the *Agrobacterium tumefaciens* strain GV3101, which was then used to transform Arabidopsis plants via the floral dip method (Clough and Bent 1998). The homozygous lines were screened based on hygromycin resistance. Confirmation of this selection was achieved by directly sequencing PCR-amplified genomic products, which were amplified with the use of primers targeting the specific region listed in Supplementary Table 1.

### Anthocyanin assay

Anthocyanins were extracted using a modified version of a previously described method (Nakata et al. 2013). Three-day-old Arabidopsis seedlings were transferred to half-strength MS medium supplemented with 1% or 3% (w/v) sucrose and grown for another three days. Samples were then extracted in 45% methanol and 5% acetic acid (v/v). The relative anthocyanin content was determined spectrophotometrically by measuring the absorbance at 520 nm and 657 nm, and the relative values were calculated accordingly.

### RNA extraction and RT-qPCR analyses

Total RNA was extracted from Arabidopsis plants using GeneAll Hybrid-R (GeneAll Biotechnology, Republic of Korea) according to the manufacturer’s instructions. First-strand cDNA was synthesized from 2 μg total RNA using M-MLV reverse transcriptase (Promega, Madison, USA). For RT-qPCR, GoTaq PCR Mix (Promega, Madison, USA) was used according to the manufacturer’s instructions. qPCR was performed using a LightCycler 2.0 system (Roche Diagnostics, Mannheim, Germany). Transcript levels were normalized to the expression of the *UBQ* gene. The primers used for RT-qPCR analysis are provided in Supplementary Table 1.

### Sucrose measurements

For sucrose extraction, 20 mg (fresh weight) of Arabidopsis rosette leaves was ground in liquid nitrogen and extracted using 80% (v/v) ethanol. The extracted samples were centrifuged at 12,000 × g for 10 minutes at 4°C, and the supernatant was filtered before analysis. Soluble sucrose content was quantified by high-performance liquid chromatography (HPLC) on a Dionex Ultimate 3000 system (Thermo Fisher, Sunnyvale, USA) equipped with a Shodex RI-101 refractive index detector (Shoko, Tokyo, Japan) at the Seoul National University NICEM. Separation was performed on a Sugar-Pak column (Waters, 300 mm × 6.5 mm) at 70°C. The mobile phase consisted of ultrapure water (Milli-Q grade) at a flow rate of 0.5 mL/min. The injection volume was set at 10 μL for each sample. Chromeleon ver. 6 software was used for data acquisition and processing. Calibration was carried out with sucrose standard (Sigma, 99.5% purity). Quantification was performed by comparing sample peak areas to those of the standard curve generated from known sucrose concentrations.

### DELLA protein stability

For the DELLA protein stability analysis, five-day-old seedlings were grown in 6-well plates with 1 mL liquid half-strength MS medium supplemented with 0% (w/v) sucrose (pH 5.7) under long-day conditions (16 h light/8 h dark, 21°C). After 3 days in LD conditions, 5% (w/v) Suc was added for an additional 3 days. Then, these seedlings were treated with 10 μM GA for 2 h, and total protein was extracted using extraction buffer containing 50 mM Tris-HCl (pH 7.5), 250 mM NaCl, 10 mM MgCl_2_, 1 mM EDTA, 0.5% Triton-X 100, 10% glycerol, 1 mM DTT, 0.2 mM PMSF, and 1× Pierce protease inhibitor (Thermo Fisher, Rockford, USA). The extracted proteins were mixed with 5× Laemmli loading buffer containing 10% SDS, 50% glycerol, 0.01% bromophenol blue, 10% beta-mercaptoethanol, and 0.3 M Tris-HCl (pH 6.8), and boiled at 95°C for 5 min. Total extracted proteins were separated by 10% SDS-PAGE gel electrophoresis, and proteins were transferred to a PVDF membrane (Immobilon^®^-P, Millipore) with a semi-dry transfer system (Trans-Blot^®^ SD, Bio-Rad, Hercules, USA). After blocking with 5% skim milk, the membrane was incubated with RGA/DELLA antibody (Agrisera, Cat No. AS11–1630, dilution 1:1000) in 5% skim milk for 2 h. Subsequenctly, the membrane was washed 3 times and incubated with secondary goat anti-rabbit-HRP (Santa Cruz, Cat No. sc-2004, dilution 1:10000) for 2 h. Subsequently, the membrane was washed 5 times in TBST (50 mM Tris, 150 mM NaCl, 0,05% Tween 20), incubated with Pierce SuperSignal^®^ West Pico chemiluminescent substrate (Thermo Scientific, Cat No. 34578) for 1 min and exposed to film.

### Gibberellins quantification

GA quantification was analyzed by the previous method (Xin et al. 2020). Briefly, 1 g of 10-day-old seedlings were ground to a find powder in liquid nitrogen, and the samples were freeze-dried for 3 days. 1 mL of solution containing 80% (v/v) methanol was added to each sample and incubated for 12 h at 4°C. The samples were centrifuged at 12,000 × g for 15 min at 4°C. The solvent was then dried down using a Speed Vac concentrator at room temperature (25°C). The dried pellets were resuspended in 100 μL of solution containing 80% (v/v) methanol and resuspended samples were immediately subjected to liquid chromatography-mass spectrometry (LC-MS) hormonal analysis (Seoul National University NICEM, Republic of Korea).

## Results

### mvk-1 mutants exhibit enhanced sucrose-induced anthocyanin accumulation

**Figure 1:**
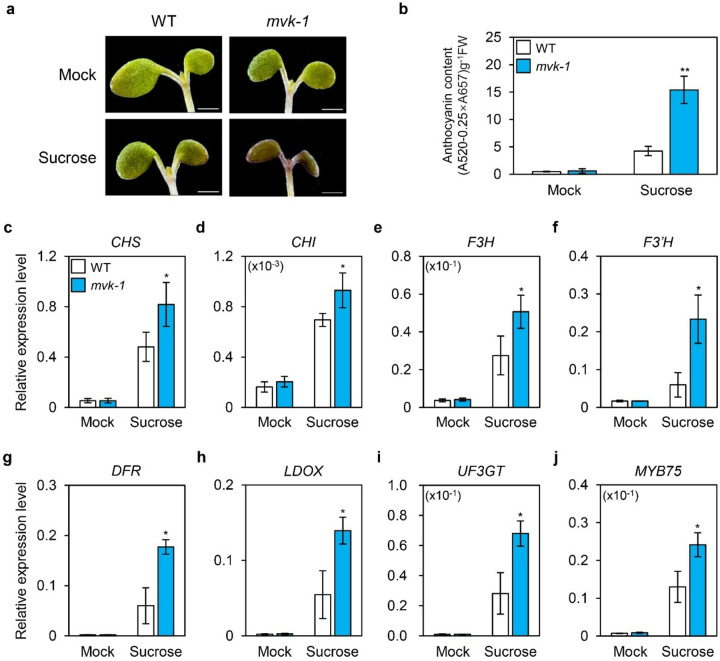
MVK is involved in sucrose-induced anthocyanin biosynthesis. **a**The *mvk-1* mutants accumulate more anthocyanins in response to sucrose treatment. 10-day-old seedlings of WT and *mvk-1* mutants grown in half strength Murashige and Skoog (MS) medium were treated with or without 3% (w/v) exogenous sucrose for 3 days. Scale bar represents 1 mm. **b**Anthocyanin content in WT and *mvk-1* mutants. Anthocyanin contents were determined by measuring the absorbance of plant extracts at 520 nm, subtracting 0.25 times the absorbance at 657 nm, and expressing the result per gram fresh weight (FW). The mean and SD were obtained from three biological replicates. Asterisks indicate significantly different values according to Student’s *t*-test (*P < 0.05, **P < 0.01). **c-j**Expression patterns of anthocyanin biosynthetic and regulatory genes. The relative expression levels of (**c**) *CHS*, (**d**) *CHI*, (**e**) *F3H*, (**f**) *F3’H*, (**g**) *DFR*, (**h**) *LDOX*, (**i**) *UF3GT*, and (**j**) *MYB75* in WT and *mvk-1*seedlings. 10-day-old seedlings grown in half-strength MS medium were treated with or without 3% (w/v) sucrose for 3 days. White bars represent WT, blue bars represent *mvk-1* mutants. Expression levels were determined by RT-qPCR and normalized to the expression of *UBQ5* reference gene. The mean and SD were obtained from four biological replicates. Asterisks indicate significantly different values according to Student’s *t*-test (*P < 0.05).

The MVA pathway plays a crucial role in the biosynthesis of a wide range of isoprenoids, including phytohormones (Pulido et al. 2012). It was reported that CK, GA, and ABA regulate the induction of anthocyanin biosynthesis by sugar in Arabidopsis (Loreti et al. 2008). This led us to examine whether mutation of MVK affects anthocyanin levels. We first investigated anthocyanin accumulation in *mvk-1* mutants. To determine the role of *MVK* in anthocyanin accumulation, 10-day-old WT and *mvk-1* plants were grown on half-strength Murashige and Skoog (MS) medium in the absence or presence of 3% sucrose for 3 days. The leaves and shoot apical meristem region of *mvk-1* mutants showed intense purple coloration after being grown on 3% sucrose (Fig. 1a). Furthermore, a pronounced anthocyanin accumulation phenotype was observed across the abaxial surface of 10-day-old *mvk-1* mutants grown on 5% sucrose medium. (Supplementary Fig. S1). There was no difference in anthocyanin content under mock conditions, whereas *mvk-1* mutants exhibited an almost 3-fold increase in anthocyanin content compared to WT under 3% sucrose conditions (Fig. 1b).

Next, to identify whether these phenotypes were due to upregulation of anthocyanin biosynthetic genes at the transcriptional level, we examined the relative expression of genes involved in the anthocyanin biosynthetic pathway (*CHS, CHI, F3H, F3’H, DFR, ANS/LDOX and UF3GT*). Under 3% sucrose conditions, *mvk-1* mutants exhibited higher expression levels of anthocyanin biosynthetic genes compared with WT (Fig. 1c–i). We also measured the relative expression level of *MYB75*, which is a transcription factor composing the MBW complex, involved in the transcriptional activation of anthocyanin biosynthetic genes (Teng et al., 2005). Interestingly, the expression level of *MYB75* was also upregulated in *mvk-1* mutants compared to WT under 3% sucrose conditions (Fig. 1j). Taken together, these findings indicate that a loss-of-function of *MVK* results in enhanced anthocyanin accumulation, likely through transcriptional activation of the MBW complex and the subsequent upregulation of key genes in the anthocyanin biosynthetic pathway.

### High sucrose accumulation in the leaves of mvk-1 mutants

**Figure 2:**
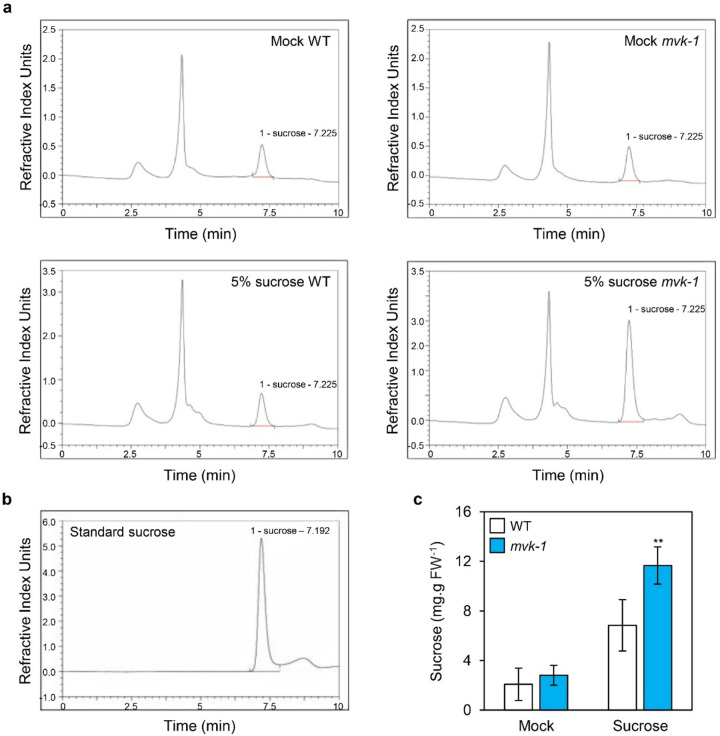
Sucrose content in the leaves of WT and *mvk-1* mutants. **a**Sucrose contents were measured from the leaves of 10-day-old WT and *mvk-1*seedlings treated with or without 5% (w/v) sucrose for 3 days. Leaf extracts were analyzed by HPLC. **b**HPLC chromatogram showing the standard sucrose peak. The retention time of sucrose was 7.192 min. **c**Sucrose contents in WT and *mvk-1*mutants. White bars represent WT, blue bars represent *mvk-1*mutants. The mean and SD were obtained from more than four biological replicates. Asterisks indicate significantly different values according to Student’s *t*-test (*P < 0.05, **P < 0.01).

Sucrose is widely recognized as a signaling molecule that induces anthocyanin biosynthesis (Yoon et al. 2021). As shown in Fig. 1, *mvk-1* mutants exhibited increased anthocyanin accumulation under high sucrose conditions. We hypothesized that this phenotype is associated with elevated sucrose levels. To confirm this hypothesis, we collected leaves from WT and *mvk-1* mutants grown under the same conditions and subjected them to the treatments with or without 5% sucrose. Sucrose content was measured using HPLC. Under mock conditions, no difference was observed in sucrose levels (i.e., peak at ~ 7.2 mins) between WT and *mvk-1* mutants. However, *mvk-1* mutants showed higher internal sucrose levels than WT in the presence of 5% sucrose (Fig. 2a, b). The standard sucrose was detected at ~ 7.2 mins (Fig. 2b). These results suggest that Arabidopsis MVK influences sucrose accumulation, potentially contributing to an enhanced anthocyanin phenotype observed in *mvk-1* mutants.

### Expression patterns of SUCs in mvk-1 mutants

**Figure 3:**
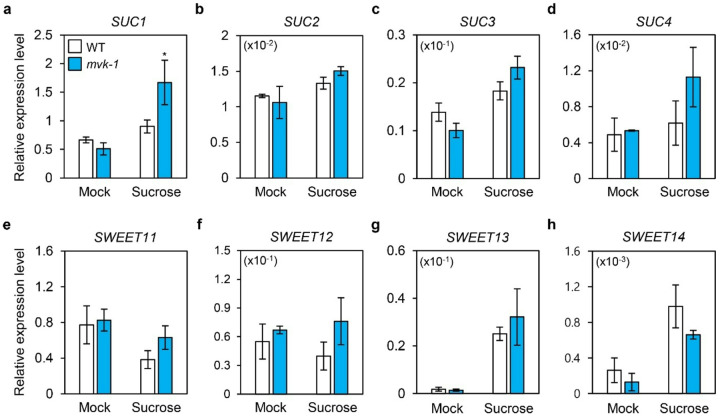
The relative expression levels of sucrose transporter genes in WT and *mvk-1* seedlings. The relative expression levels of (**a**) *SUC1*, (**b**) *SUC2*, (**c**) *SUC3*, (**d**) *SUC4*, (**e**) *SWEET11*, (**f**) *SWEET12*, (**g**) *SWEET13*, and (**h**) *SWEET14* in 10-day-old seedlings grown in half-strength MS medium were treated with or without 3% (w/v) sucrose for 1 day. White bars represent WT, blue bars represent *mvk-1* mutants. Transcript levels were determined by RT-qPCR and normalized to the expression of *UBQ5* reference gene. The mean and SD were obtained from four biological replicates. Asterisks indicate significantly different values according to Student’s *t*-test (*P< 0.05).

Sucrose transporters are known to play a central role in regulating sucrose levels in plants (Julius et al. 2017). Since *mvk-1* mutants exhibited increased sucrose accumulation under high exogenous sucrose treatments (Fig. 2c), we investigated whether this phenotype was associated with altered expression of sucrose transporter genes. In order to measure expression of *SUC* genes, we compared their transcript levels between WT and *mvk-1* seedlings under high-sucrose conditions. The expression of *SUC1* was increased in *mvk-1* mutants under high-sucrose conditions (Fig. 3a), whereas that of *SUC2, SUC3*, and *SUC4* showed no significant change regardless of sucrose concentrations (Fig. 3b–d).

In addition to *SUC* genes, we also examined the *SWEET* (*Sugars Will Eventually be Exported Transporters*) genes encoding sugar transporters, which function as unidirectional uniporters mediating sucrose efflux across the plasma membrane and tonoplast (Ji et al. 2022). Among the 17 Arabidopsis *SWEET* genes, *SWEET11, SWEET12, SWEET13*, and *SWEET14* are known to participate in sucrose transport. However, no significant differences in the expression of these genes were detected between WT and *mvk-1* mutants under high-sucrose conditions (Fig. 3e–h). Taken together, these results indicated that the mutation of *MVK* specifically alters the expression of *SUC1*, while other sucrose transporters (*SUC2, SUC3, SUC4*, and *SWEET11–14*) remain unaffected.

### MVK genetically affects SUC1-mediated anthocyanin accumulation

Arabidopsis SUC1 is plasma membrane-localized sucrose/H^+^ symporters with distinct expression patterns, where SUC1 mediates local sucrose uptake in roots, trichomes, and pollen (Sauer and Stolz 1994; Sivitz et al. 2008; Stadler and Sauer 2019). Since exogenous sucrose strongly induces anthocyanin biosynthesis and SUC1 primarily functions in sucrose uptake in roots, we hypothesized that *SUC1* expression is a downstream target of MVK. To investigate whether MVK regulates anthocyanin accumulation through *SUC1*, we generated the CRISPR/Cas9-mediated *suc1–5* mutants in WT background, harboring a single base insertion that resulted in a premature stop codon in the *SUC1* coding region (Supplementary Fig. S2). Additionally, the *mvk-1 suc1–5* double mutants were created by introducing the *SUC1* CRISPR/Cas9 construct into the *mvk-1* background to examine their genetic interaction. Sequencing analysis confirmed a single base insertion in *SUC1*, which is identical to the *suc1–5* mutant allele (Supplementary Fig. S2b).

**Figure 4:**
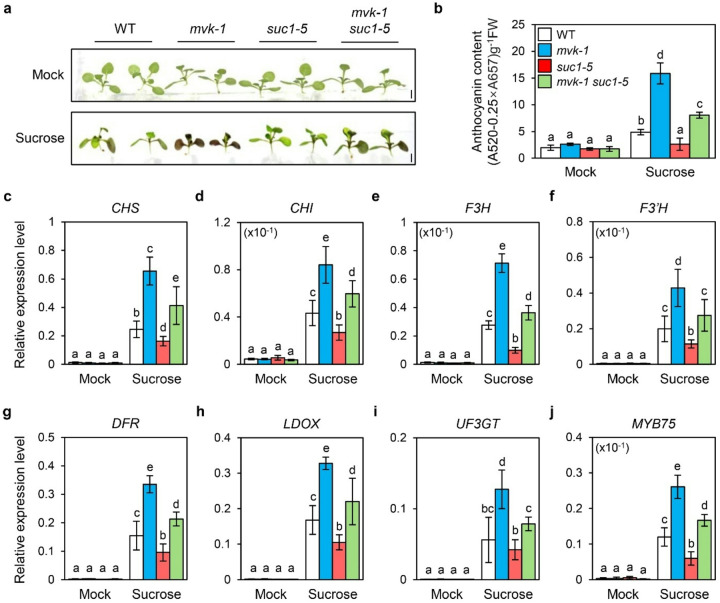
Expression patterns of anthocyanin biosynthetic and regulatory genes. **a**Anthocyanin accumulation with or without 3% (w/v) sucrose treatment in WT, *mvk-1, suc1–5*, and *mvk-1 suc1–5* mutants. 10-day-old seedlings of WT, *mvk-1, suc1–5*, and *mvk-1 suc1–5* mutants grown in half strength MS medium were treated with or without 3% (w/v) sucrose for 3 days. Scale bar represents 0.1 cm. **b**Anthocyanin contents in WT, *mvk-1, suc1–5*, and *mvk-1 suc1–5* mutants. Anthocyanin contents were determined by measuring the absorbance of plant extracts at 520 nm, subtracting 0.25 times the absorbance at 657 nm, and expressing the result per gram fresh weight (FW). **c-j**The relative expression levels of (**c**) *CHS*, (**d**) *CHI*, (**e**) *F3H*, (**f**) *F3’H*, (**g**) *DFR*, (**h**) *LDOX*, (**i**) *UF3GT*, and (**j**) *MYB75* in WT, *mvk-1, suc1–5*, and *mvk-1 suc1–5* seedlings. 10-day-old whole seedlings grown in half-strength MS medium were treated with or without 3% (w/v) sucrose for 3 days. The white, blue, red, and green bars represent WT, *mvk-1, suc1–5*, and *mvk-1 suc1–5* mutants, respectively. Expression levels were determined by RT-qPCR and normalized to the expression of *UBQ5* reference gene. The mean and SD were obtained from four biological replicates. Different letters indicate significantly different values according to a one-way ANOVA followed by Duncan’s least significant range test (*P < 0.05).

Consistent with previous studies (Sivitz et al. 2008), *suc1–5* mutants exhibited less anthocyanin accumulation under 3% sucrose treatment compared to WT (Fig. 4a, b). Interestingly, *mvk-1 suc1–5* double mutants showed intermediate anthocyanin levels, higher than *suc1–5* but lower than *mvk-1* mutants (Fig. 4a, b). At the transcriptional level, anthocyanin biosynthetic genes were markedly downregulated in *suc1–5* mutants, but significantly upregulated in *mvk-1 suc1–5* double mutants compared with WT under sucrose treatment (Fig. 4c–i). Similarly, *MYB75* expression level was reduced in suc1–5 mutants, whereas it was induced in *mvk-1 suc1–5* double mutants (Fig. 4j). Together, these results suggested that MVK regulates anthocyanin accumulation partially by downregulating *SUC1* expression, while also acting through additional SUC1-independent pathways.

### MVK regulates DELLA stability via GA biosynthesis

**Figure 5:**
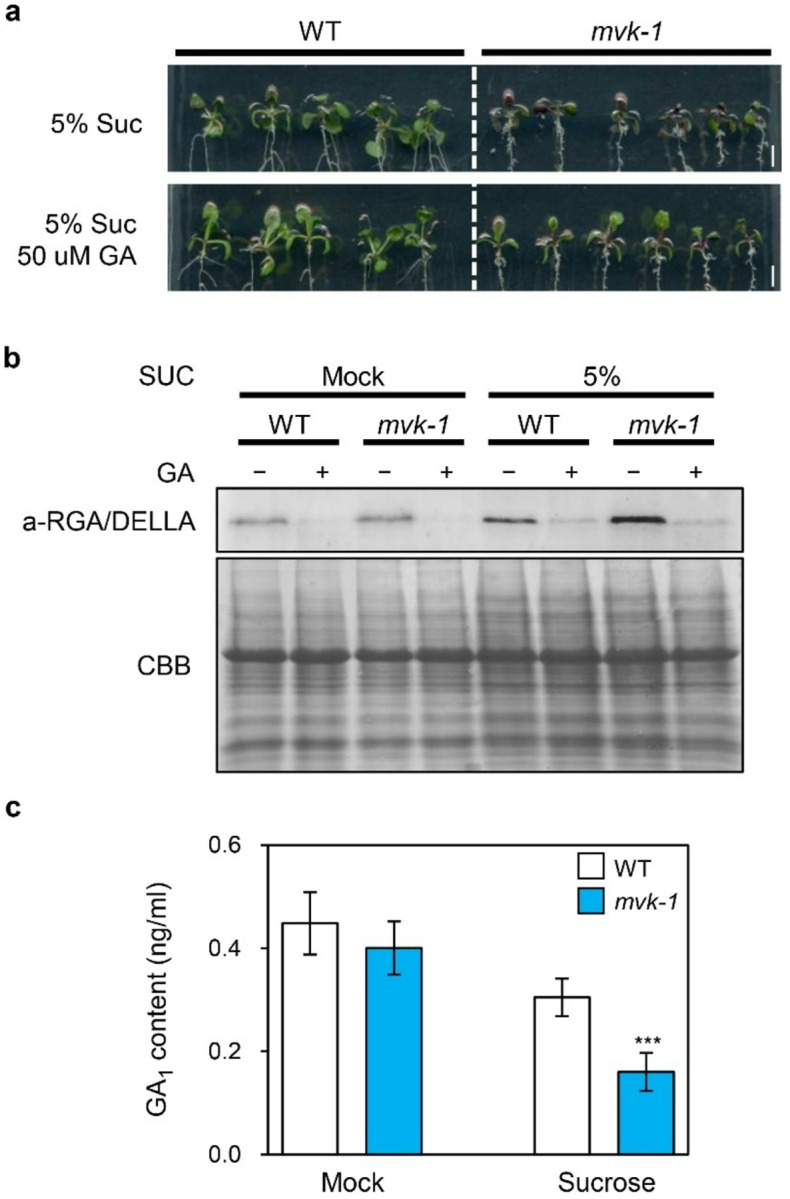
The *mvk-1* mutants accumulate lower levels of gibberellins (GA_1_) than WT. **a** Anthocyanin accumulation under 5% (w/v) sucrose with or without 50 μM GA treatments in WT and *mvk-1* mutants. Scale bar represents 0.3 cm. **b** Measurement of DELLA protein levels. WT and *mvk-1* mutants were grown under with or without 3% (w/v) sucrose treatments with or without GA. Total DELLA proteins were detected by immunoblotting with an anti-RGA/DELLA antibody. Protein loading was visualized by Coomassie brilliant blue (CBB) staining. **c** GA_1_ quantification by LC-MS. 14-day-old whole seedlings of WT and *mvk-1* mutants grown in half-strength MS medium were treated with or without 3% (w/v) sucrose for 3 days. The mean and SD were obtained from more than four biological replicates. Asterisks indicate significantly different values according to Student’s *t*-test (*P < 0.05, **P < 0.01, ***P < 0.001).

Interestingly, *mvk-1 suc1–5* double mutants showed significantly higher anthocyanin accumulation compared to *suc1–5* mutants under sucrose treatment (Fig. 4a, b). Since the MVA and MEP pathway synthesizes isoprenoid precursors essential for phytohormones, such as brassinosteroids, CK, GA, and ABA. (Pulido et al. 2012), and GA suppresses sucrose-induced anthocyanin accumulation (Loreti et al. 2008), we hypothesized that MVK may regulate anthocyanin accumulation not only via a SUC1-mediated pathway but also through GA biosynthesis. To confirm this, we compared the anthocyanin accumulation of WT and *mvk-1* mutants under 5% sucrose with or without 50 μM GA treatments. As shown in Fig. 5a, the accumulation of anthocyanins in *mvk-1* mutants under 5% sucrose treatment was attenuated by GA application. Since GA represses sucrose signaling by promoting degradation of DELLA proteins (Li et al. 2014), we measured the level of DELLA proteins in WT and *mvk-1* mutants under sucrose treatment with or without GA treatments. Remarkably, *mvk-1* mutants showed higher levels of DELLA proteins compared to WT, regardless of GA treatment under each sucrose conditions (Fig. 5b). Since the mutants exhibited elevated levels of DELLA protein, we investigated whether this was associated with altered GA content. Analysis by LC-MS revealed that GA_1_ levels in the *mvk-1* mutants were significantly lower than those in WT (Fig. 5c).

Previous studies demonstrated that GA biosynthetic genes *Gibberellin 3-oxidase 1* (*GA3ox1*) and *Gibberellin 20-oxidase 1* (*GA20ox1*) exhibit increased expression under GA-deficient conditions, consistent with feedback regulation mechanisms (Fukazawa et al. 2014). Similarly, our results revealed significant upregulation of *GA3ox1* and *GA20ox1* expression in *mvk-1* mutants, whereas their expression was downregulation in *suc1–5* mutants compared to WT (Supplementary Fig. S4). Notably, *mvk-1 suc1–5* mutants showed moderate expression levels, significantly higher than *suc1–5* mutants but lower than *mvk-1* mutants. Taken together, our results suggest that reduced GA biosynthesis in *mvk-1* mutants impedes the degradation of DELLA proteins, resulting in higher anthocyanin accumulation compared to WT.

## Discussion

### Loss of MVK enhances sucrose-specific induction of anthocyanin biosynthetic pathway

Anthocyanin accumulation is closely linked to the availability of sucrose, since sucrose transporters such as SUCs and SWEETs import extracellular sucrose, thereby inducing the anthocyanin biosynthesis. It was previously reported that several kinases regulate sucrose transporters. For example, Sucrose-Induced Receptor Kinase 1 (SIRK1) phosphorylates and thereby activates several membrane proteins including SWEET11 under sucrose-specific osmotic response (Wu et al. 2013). Furthermore, Wall-Associated Kinase Like 8 (WAKL8) phosphorylates SUC2, thereby increasing its transport activity (Xu et al. 2020). However, although kinase-mediated regulation of sucrose transporters has been demonstrated, no studies have yet reported a mechanism by which such kinase-dependent modulation of sucrose transporters directly influences anthocyanin biosynthesis.

In this study, we showed that the knockout mutation of *MVK* enhances sucrose-specific anthocyanin accumulation (Fig. 1). The *mvk-1* mutants showed increased expression of anthocyanin biosynthetic genes, along with higher transcript levels of *MYB75*, a key transcriptional regulator of anthocyanin biosynthetic genes (Fig. 1j). Measurement of sucrose contents in leaves revealed that *mvk-1* mutants accumulate higher levels of sucrose under high-sucrose conditions compared to WT (Fig. 2). Consistently, *mvk-1* mutant plants showed significantly increased expression of *SUC1*, indicating that MVK negatively regulates *SUC1* expression. Furthermore, *mvk-1 suc1–5* double mutants showed significantly higher anthocyanin accumulation than the *suc1–5* mutants, indicating that the enhanced anthocyanin phenotype of *mvk-1* mutants is at least partially dependent on *SUC1*. DELLA proteins levels remained higher in *mvk-1* mutants than WT under sucrose treatment. LC-MS analysis also indicated that *mvk-1* mutants had lower GA_1_ levels compared to WT.

**Figure 6:**
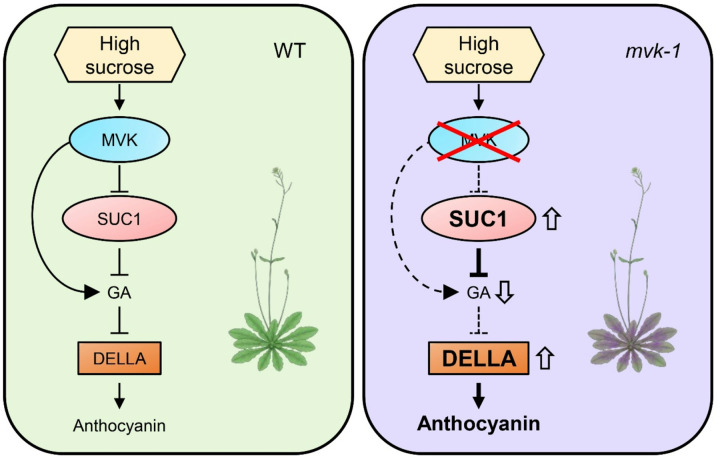
MVK regulates anthocyanin synthesis by modulating *SUC1* expression and GA biosynthesis. Schematic model shows the role of MVK in regulating anthocyanin biosynthesis. MVK represses *SUC1* expression, which in turn downregulates sucrose levels, leading to altered GA levels, both directly and indirectly. Reduced GA levels stabilize DELLA proteins, thereby activating the MBW complex and enhancing anthocyanin accumulation in Arabidopsis.

Taken together, these results strongly suggest that *MVK* regulates anthocyanin accumulation in plants by modulating *SUC1* expression. In addition, MVK controls GA levels, and the absence of MVK activity leads to reduced GA_1_ content and increased DELLA protein stability, which further promotes the expression of anthocyanin biosynthetic genes. These regulatory mechanisms contribute to enhanced anthocyanin accumulation observed in *mvk-1* mutants (Fig. 6).

### The MVA pathway regulates anthocyanin accumulation via GA biosynthesis

Anthocyanin accumulation is tightly controlled by the interplay between sucrose and phytohormones. Auxin and cytokinin promote anthocyanin accumulation through transcriptional activation and antioxidant regulation, whereas ethylene exerts context-dependent effects (Bhaskar et al. 2021; Chandler 2016; Ni et al. 2021). Abscisic acid strongly induces anthocyanin biosynthesis under stress conditions, while GA consistently acts as a negative regulator (Karppinen et al. 2018; Loreti et al. 2008). Moreover, sucrose signaling was shown to interact with several hormones, including IAA, ABA, MeJA, and SA, but is antagonized by GA (Li et al. 2014).

Our results expand this framework by demonstrating that MVK, a key enzyme in the MVA pathway, influences anthocyanin accumulation through GA biosynthesis. The *mvk-1 suc1–5* double mutants accumulated more anthocyanin than *suc1–5* mutants (Fig. 4a, b). Moreover, the double mutants also exhibited reduced root length (Supplementary Fig. S3). These observations suggest the existence of another regulatory pathway compensating for the loss of SUC1-mediated sucrose signaling. Notably, the dwarfism observed in both *mvk-1* and *mvk-1 suc1–5* mutants (Supplementary Figs. S1, S3) aligns with established roles of the MVA pathway in isoprenoid biosynthesis, which supplies precursors for GA synthesis (Cho et al. 2022).

In *mvk-1* mutants, reduced GA_1_ levels and elevated DELLA protein accumulation (Fig. 5c) alleviate GA-mediated repression of sucrose signaling, thereby enabling enhanced anthocyanin accumulation in *mvk-1 suc1–5* mutants even in the absence of *SUC1* (Fig. 4). Furthermore, exogenous GA treatment rescued the hyperaccumulation of anthocyanin phenotype in *mvk-1* mutants (Fig. 5a). Disruption of MVK reduces the amount of GGPP-derived GA precursors, thereby leading to the stabilization of DELLA proteins. These findings highlight how GA hormonal signals linked to the MVA pathway are intertwined in fine-tuning plant secondary metabolism, such as anthocyanin biosynthesis. Our study uncovers a previously unrecognized role of MVK in repressing the expression of *SUC1* (Fig. 3a). This transcriptional repression connects the MVA-GA signaling module to sucrose transport, suggesting that MVK serves as an integrative regulator bridging hormonal and metabolic cues. Our findings therefore propose a broader role for the MVK-SUC1 regulatory axis in anthocyanin biosynthesis. Since anthocyanin accumulation is tightly controlled by sucrose availability and stress-induced signaling pathways, the MVK-SUC1 connection may represent a critical node that coordinates primary metabolism, signaling networks, and secondary metabolism. This integration could enable plants to balance growth and stress adaptation by modulating anthocyanin levels.

## Supplementary Files

This is a list of supplementary files associated with this preprint. Click to download.

- SupplementaryMaterial.docx

## Data Availability

The datasets generated during and/or analyzed during the current study are available from the corresponding author on reasonable request.

## Key Message

Arabidopsis MVK negatively regulates sucrose-induced anthocyanin accumulation by modulating SUC1-mediated sucrose transport and gibberellin homeostasis.
